# Early predictors of severe COVID‐19 among hospitalized patients

**DOI:** 10.1002/jcla.24177

**Published:** 2021-12-23

**Authors:** Qiongrui Zhao, Youhua Yuan, Jiangfeng Zhang, Jieren Li, Wei Li, Kunshan Guo, Yanchao Wang, Juhua Chen, Wenjuan Yan, Baoya Wang, Nan Jing, Bing Ma, Qi Zhang

**Affiliations:** ^1^ Centre of Clinical Research Service Henan Provincial People’s Hospital People’s Hospital of Zhengzhou University People’s Hospital of Henan University Zhengzhou China; ^2^ Department of Clinical Microbiology Henan Provincial People’s Hospital People’s Hospital of Zhengzhou University People’s Hospital of Henan University Zhengzhou China; ^3^ Department of Critical Care Medicine Huaibin County People’s Hospital Huaibin China; ^4^ Department of Infectious Disease Henan Provincial People’s Hospital People’s Hospital of Zhengzhou University People’s Hospital of Henan University Zhengzhou China; ^5^ Department of Clinical Microbiology Xuchang Municipal Central Hospital Xuchang China; ^6^ Department of Clinical Microbiology Hebi Infectious Disease Hospital Hebi China; ^7^ Department of Clinical Microbiology Xinyang Municipal First People’s Hospital Xinyang China

**Keywords:** cohort study, COVID‐19, prediction model, predictor

## Abstract

**Background:**

Limited research has been conducted on early laboratory biomarkers to identify patients with severe coronavirus disease (COVID‐19). This study fills this gap to ensure appropriate treatment delivery and optimal resource utilization.

**Methods:**

In this retrospective, multicentre, cohort study, 52 and 64 participants with severe and mild cases of COVID‐19, respectively, were enrolled during January‐March 2020. Least absolute shrinkage and selection operator and binary forward stepwise logistic regression were used to construct a predictive risk score. A prediction model was then developed and verified using data from four hospitals.

**Results:**

Of the 50 variables assessed, eight were independent predictors of COVID‐19 and used to calculate risk scores for severe COVID‐19: age (odds ratio (OR = 14.01, 95% confidence interval (CI) 2.1–22.7), number of comorbidities (OR = 7.8, 95% CI 1.4–15.5), abnormal bilateral chest computed tomography images (OR = 8.5, 95% CI 4.5–10), neutrophil count (OR = 10.1, 95% CI 1.88–21.1), lactate dehydrogenase (OR = 4.6, 95% CI 1.2–19.2), C‐reactive protein OR = 16.7, 95% CI 2.9–18.9), haemoglobin (OR = 16.8, 95% CI 2.4–19.1) and D‐dimer levels (OR = 5.2, 95% CI 1.2–23.1). The model was effective, with an area under the receiver‐operating characteristic curve of 0.944 (95% CI 0.89–0.99, *p* < 0.001) in the derived cohort and 0.8152 (95% CI 0.803–0.97; *p* < 0.001) in the validation cohort.

**Conclusion:**

Predictors based on the characteristics of patients with COVID‐19 at hospital admission may help predict the risk of subsequent critical illness.

## Graphical abstract

This study aims to conduct on early laboratory biomarkers to identify patients with severe coronavirus disease (COVID‐19) .Furtherly, to ensure appropriate treatment delivery and optimal resource utilisation.

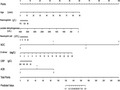

## 1 INTRODUCTION

The novel coronavirus disease (COVID‐19) has rapidly spread worldwide. As of 1 August 2021, the World Health Organization reported a total of 198,754,713 COVID‐19 cases globally, with an average mortality of 6.57%. The clinical spectrum of COVID‐19 pneumonia ranges from asymptomatic carriers to mild and critically ill cases, and there is a high mortality rate among patients with severe disease.^1^ Therefore, early detection of patients who are likely to develop critical illness is of great importance, and it may help in proper care delivery and optimal use of limited resources.^2^ Some studies have reported that predictors of the development of severe illness and diseases related to COVID‐19 include older age, neutrophilia, organ dysfunction and coagulopathy.^3^, ^4^, ^5^, ^6^ However, limited research has been conducted on early laboratory biomarkers to identify patients with severe disease. In this study, we aimed to construct an outcome prediction model based on laboratory testing parameters and other predictors identified in a retrospective cohort study of Chinese patients with COVID‐19 in central China. The model can help identify patients likely to develop critical illness at hospital admission.

## 2 MATERIALS AND METHODS

### 2.1 Research overview

The derivation cohort consisted of patients who visited Henan Provincial People's Hospital (Henan) from January to March 2020. This is a tertiary teaching hospital in central China, with 5,000 hospital beds, including 300 intensive care unit (ICU) beds. The annual volume of infectious patients admitted to this hospital is approximately 2,400. The validation cohort consisted of patients who visited Xuchang Municipal Central Hospital (Xuchang), Hebi Infectious Disease Hospital (Hebi), Huaibin County People's Hospital (Huaibin) and Xinyang Municipal First People's Hospital (Xinyang) during the same period. Patients in the derivation and validation cohorts were selected according to the inclusion and exclusion criteria in the Diagnostic and Treatment Guidelines for COVID‐19 issued by the Chinese National Health Committee (Version 7).^7^ The laboratory parameters of patients from the derivation and validation cohorts were detected using the same testing methods, equipment and reagents. Patients were defined to have severe COVID‐19 if they met one of the following criteria: (1) respiratory distress with respiratory frequency ≥30/min; (2) pulse oximeter oxygen saturation ≤93% at rest and (3) oxygenation indexes (artery partial pressure of oxygen/inspired oxygen fraction) ≤300 mmHg. These hospitals were designated as COVID‐19 treatment centres by the Health Committee of Henan Province in January 2020.

### 2.2 Data collection and clinical assessment

COVID‐19 diagnoses were confirmed using positive real‐time reverse‐transcription polymerase chain reaction assays of nasal and pharyngeal swab specimens, sputum or stool samples or specific immunoglobulin (Ig) M or Ig G antibody testing of the serum.^7^ A team of experienced infectious disease clinicians reviewed and cross‐checked the data. Two clinicians independently checked each record. The criterion for variable selection was in accordance with previous studies and related specifically to patients with severe disease.^8^, ^9^ We included all patients with available data on clinical status during hospitalization (laboratory findings, clinical symptoms and signs, illness severity and discharge status). After assessing the data, it was found that the data from Henan Provincial People's Hospital were more complete than those from the other four hospitals; therefore, the data from Henan Provincial People's Hospital were used for the derivation cohort study, while the data from the other four hospitals were used in the validation cohort study.

### 2.3 Outcome definitions

We defined the severity of COVID‐19 (severe v. mild) based on the Diagnostic and Treatment Guidelines for COVID‐19 issued by the Chinese National Health Committee (Version 7).^7^

### 2.4 Potential predictive variables

In total, 50 potential predictive variables were collected: 30 related to clinical data and 20 to laboratory testing parameters. Clinical data included the following patient characteristics at hospital admission: clinical signs and symptoms, imaging results, demographic variables and medical history. Demographic variables included age, gender, exposure to Hubei Province (including travel history to Hubei or contact with confirmed patients) and time between onset of symptoms and admission. Medical history included the number of comorbidities, that is, presence of chronic obstructive pulmonary disease, diabetes, hypertension, coronary artery disease, hepatitis B and hypoalbuminemia. Clinical signs and symptoms included categorical and continuous variables: initial body temperature, fever, cough, expectoration, fatigue and diarrhoea. Imaging results included abnormal chest computed tomography (CT), as well as the number of abnormalities and the site(s) of CT abnormalities. Upon admission to the hospital, 20 laboratory parameters were collected, including routine indexes of blood examination, such as lymphocyte, platelet and neutrophil counts, as well as haemoglobin and C‐reactive protein levels. Inflammatory cytokines, including procalcitonin, interleukin (IL)‐6, IL‐10, serum ferritin protein, coagulation function indicators (including D‐dimer and fibrinogen levels) and liver function indicators (such as lactate dehydrogenase levels), were also included. Further, we collected data on immune function indicators, including B‐lymphocyte count, T‐lymphocyte count, natural killer cell count and Ig M and Ig G antibody titres.

### 2.5 Variable selection and model construction

In the derivation cohort, 65 patients (Table 1) were included in the variable selection and risk score development. As described herein, 50 variables were entered in the selection process. Least absolute shrinkage and selection operator (LASSO) regression were applied to minimize the potential collinearity of variables measured from the same patient and over‐fitting of variables. Imputation for missing variables was considered if missing values were less than 20%. We used predictive mean matching to impute numeric features, logistic regression to impute binary variables and Bayesian polytomous regression to impute factor features. We used penalized LASSO regression for multivariable analyses, augmented with 10‐fold cross‐validation for internal validation. This is a logistic regression model that penalizes the absolute size of the coefficients of a regression model based on the value of λ. With larger penalties, the estimates of weaker factors shrink towards zero, so that only the strongest predictors remain in the model. The most predictive covariates were selected by the minimum (λ min). The R package ‘glmnet’ statistical software (R 4.0.3 version) was used to perform the LASSO regression. Subsequently, variables identified by the LASSO regression analysis were entered into binary logistic regression models, and those that were consistently statistically significant were used as early predictors and in the construction of the risk score, which was then used to construct a risk score card calculator. Two‐sided *p*‐values of < 0.05 were considered statistically significant.

**TABLE 1:** Demographic and clinical characteristics in severe and mild patients as well as carriers of COVID‐19 in the derivation cohort^a^

Characteristics	Patients with severe disease (*n* = 36) *n* (%)	Patients without severe disease (*n* = 29) *n* (%)
Age, years, mean (SD)	60.4 (16.5)	38.8 (14.4)
Males	16 (45.7)	20 (69.0)
Time of admission from symptom onset, days, mean (SD)	11.6 (8.4)	6.4 (2.2)
Length of hospitalization, days, mean (SD)	11 (6.6)	8.8 (4.0)
Time of positive PCR result, days, mean (SD)	13.0 (8.3)	4.2 (3.6)
Time of symptom onset, days, mean (SD)	27.1 (9.8)	13.3 (2.3)
30‐day mortality	6 (17.1)	0 (0)
Hospitalization in ICU	7 (20)	0 (0)
History of travel to Hubei Province during COVID‐19 outbreak	15 (41.7)	14 (48.3)
Contact with confirmed patients	17 (47.2)	6 (20.7)
Fever	28 (80)	15 (51.7)
Dry cough	33 (94.3)	18 (62.1)
Fatigue	13 (37.1)	6 (20.7)
Diarrhoea	6 (17.1)	10 (34.5)
Shortness of breath	12 (34.3)	1 (3.4)
Expectoration	10 (28.6)	1 (3.4)
Any	28 (80)	5 (17.2)
0	7 (20)	22 (75.9)
1	8 (22.9)	4 (13.8)
2	10 (28.6)	2 (6.9)
≥ 3	8 (22.9)	1 (3.4)
COPD	2 (5.7)	1 (3.4)
Diabetes	16 (45.7)	1 (3.4)
Hypertension	10 (28.6)	3 (10.3)
Cardiovascular disease	6 (17.1)	0 (0)
Hepatitis B	1 (2.9)	2 (6.9)
Hypoalbuminemia	8 (22.9)	1 (3.4)
0	0 (0)	10 (34.5)
1	2 (5.8)	7 (24.1)
2	33 (94.3)	12 (41.4)
White cell count, ×10^9/^L	7.22 (4.42)	4.39 (1.66)
Neutrophil cell count, ×10^9/^L	5.71 (4.25)	2.63 (1.37)
Lymphocyte count, ×10^9^/L	1.15 (0.56)	1.38 (0.53)
Eosinophil count, ×10^9^/L	0.063 (0.02)	0.052 (0.02)
IL‐10 (ng/ml)	5.49 (1.92)	3.28 (1.80)
IL‐6 (ng/ml)	61.63 (27.36)	6.31 (2.47)
Fibrinogen (g/L)	3.91 (2.29)	3.02 (1.29)
D‐dimer level (mg/L)	3.51 (1.23)	0.53 (0.14)
Natural killer count (cells/μl)	177 (35.41)	222.7 (31.0)
T‐lymphocyte count (cells/μl)	765.8 (539.4)	1062 (501.1)
B‐lymphocyte count (cells/μl)	175 (157.3)	203.8 (169.2)
Haemoglobin (g/L)	106.9 (20.73)	138.4 (17.8)
Red cell count, ×10^9^/L	3.55 (0.70)	4.56 (0.56)
Platelet count, ×10^9^/L	256.7 (128.4)	196.2 (66.6)
Ferritin (ng/ml)	643.3 (105.0)	244.5 (61.5)
C‐reactive protein (mg/L)	52.44 (10.32)	15.69 (4.94)
Procalcitonin (ng/ml)	0.39 (0.17)	0.06 (0.02)
Lactate dehydrogenase (U/L)	272.8 (159.7)	182.8 (65.9)
Titre of IgM	9.0 (6.7)	4.1 (1.2)
Titre of IgG	8.98 (1.61)	9.47 (2.2)

Abbreviations: COPD, chronic obstructive pulmonary disease; COVID‐19: coronavirus disease; CT, computed tomography; ICU, intensive care unit; IL, interleukin; PCR, polymerase chain reaction; SD, standard deviation.

^a^ Total number of patients with available data.

### 2.6 Assessment of accuracy

The accuracy of the COVID risk score was assessed using the area under the receiver‐operating characteristic curve (AUC). For internal validation of the accuracy estimates and to reduce overfit bias, we used 200 bootstrap resamples. The calibration curve was plotted, and the Brier score was calculated to determine its accuracy evaluation. The Brier calculation formula is Brier = (Y‐p)2, where Y is the actual outcome variable (0 or 1), and p is the predictive probability calculated by the model. The closer the Brier score is to 0, the more accurate the model is. *p*‐values < 0.05 were considered statistically significant. Figures were drawn using GraphPad Prism version 8.0 (GraphPad Software Inc.) or R software version 4.0.3 (R Foundation for Statistical Computing).

## 3 RESULTS

### 3.1 Clinical features of patients in the derived and validated cohorts

In total, 116 patients with COVID‐19 were enrolled in the study, including 65 patients from Henan Provincial People's Hospital in the derivation cohort (Table 1). In the validation cohort (Table 2), 29 patients from Xuchang Municipal Central Hospital, 12 from Hebi Infectious Disease Hospital, 5 from Huaibin County People's Hospital and 5 from Xinyang Municipal First People's Hospital were included. In the derivation cohort, 55.4% (36/65) were in the severe group, while the remaining 44.6% (29/65) were in the mild group. The validation cohort included 51 patients with a mean (standard deviation) age of 48.2 (15.2) years; of these, 38 (74.5%) were male, and 12 (23.5%) had at least one coexisting condition. Critical illness eventually developed in 16 (31.4%) of these 51 patients but no patient died. Variables used for the validation cohort are shown in Table 2. Data on some laboratory variables (including IL‐6 and T lymphocyte count) were missing in the validation cohort, either because the relevant tests could not be conducted or owing to limits on the laboratory staff's authorization to view medical records at the four hospitals.

**TABLE 2:** Demographics and clinical characteristics of patients in the validation cohort^a^

Characteristic	Patients with severe disease (*n* = 16) Mean (SD)	Patients without severe disease (*n* = 35) Mean (SD)
Age (years)	62.1 (17.7)	42.3 (16.2)
Male, *n* (%)	12 (75)	20 (57.1)
White cell count, ×10^9/^L	6.96 (4.99)	5.43 (2.57)
Neutrophil cell count, ×10^9/^L	5.56 (4.82)	3.50 (2.53)
Lymphocyte count, ×10^9^/L	0.93 (0.46)	1.46 (0.91)
Eosinophil count, ×10^9^/L	0.089 (0.05)	0.032 (0.01)
D‐dimer level (mg/L)	4.18 (1.75)	0.42 (0.07)
Haemoglobin (g/L)	121.9 (21.5)	136.7 (17.3)
Red cell count, ×10^9^/L	3.94 (0.78)	4.51 (0.58)
Platelet count, ×10^9^/L	210 (63.27)	189.1 (66.4)
C‐reactive protein (mg/L)	38.9 (11.80)	21.8 (5.13)
Procalcitonin (ng/ml)	0.19 (0.07)	0.07 (0.01)
Lactate dehydrogenase (U/L)	305.3 (74.9)	277.5 (38.0)

^a^ Data are presented as mean (standard deviation [SD]) or as *n* (%), where n is the number of patients with available data.

### 3.2 Predictors of severe disease

**FIGURE 1:**
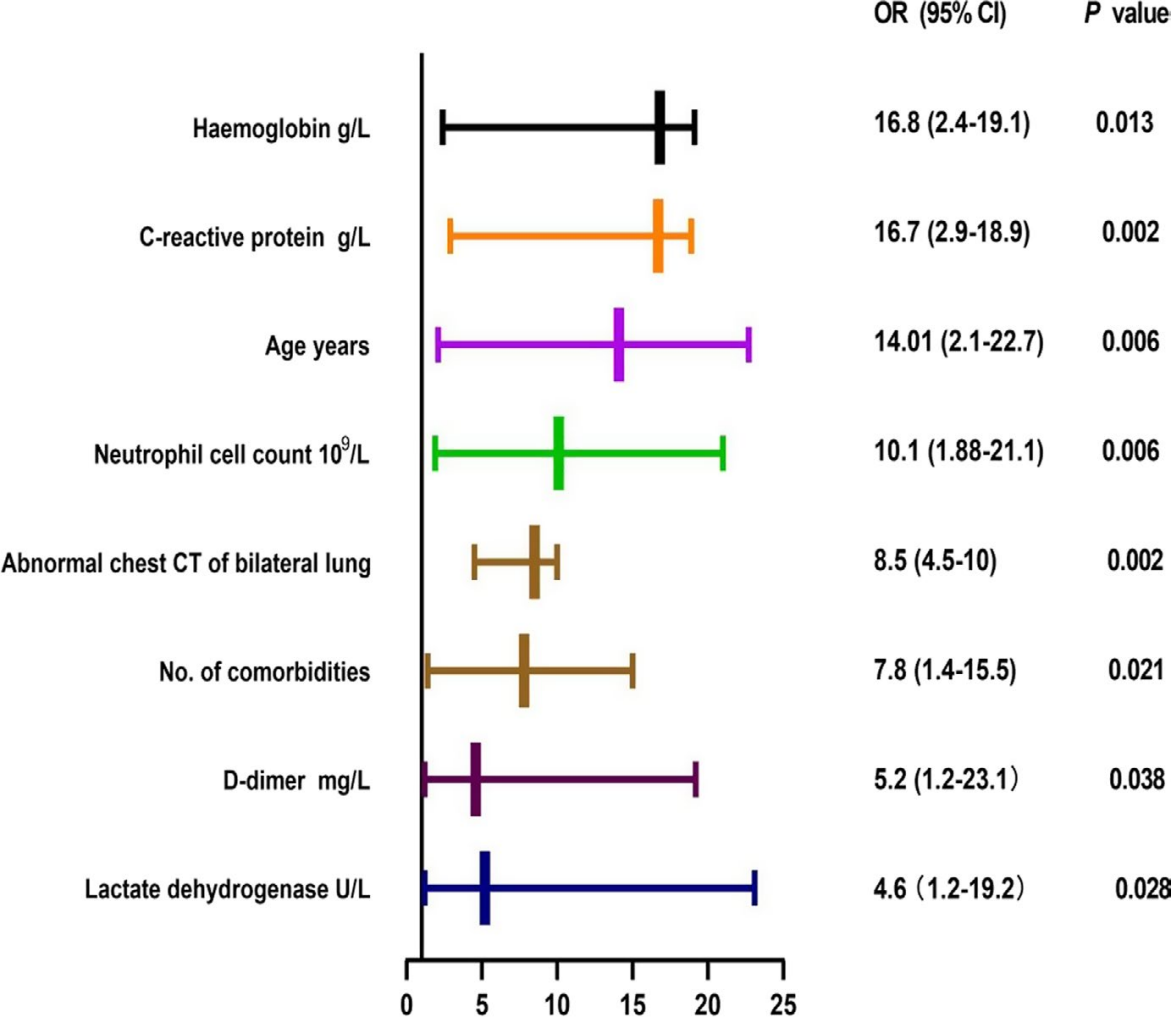
Predictors of severe COVID‐19. In the multivariate logistic regression analysis, out of the eight measured predictors, haemoglobin (g/l), C‐reactive protein (g/L) and age (years) were the top three predictors. COVID‐19, coronavirus disease; CT, computed tomography

Predictors of severe COVID‐19 were analysed using data from the derivation cohort. Fifty variables measured at initial hospital admission (Table 1) were included in the LASSO regression. After LASSO regression selection (Figure S1), 29 variables remained significant predictors of critical illness, including clinical features and blood test results, CT bilateral lung abnormality, age, neutrophil count, procalcitonin, number of comorbidities, lactate dehydrogenase, IL‐6 and D‐dimer levels, T‐lymphocyte counts, haemoglobin and other laboratory parameters. Based on the analysis of the LASSO regression, 29 variables were included in the binary logistic regression model. Eight variables with *p* < 0.05 in the multivariate binary forward stepwise logistic regression were retained in the final model (Figure 1 and Figure S2). Age (years) (odds ratio (OR) = 14.01, 95% confidence interval (CI) 2.1–22.7), number of comorbidities (OR = 7.8, 95% CI 1.4–15.5), bilateral abnormalities on chest CT (OR = 8.5, 95% CI 4.5–10) and lactate dehydrogenase level (U/L) (OR = 4.6, 95% CI 1.2–19.2) were found to be predictors of severe COVID‐19 (*p* < 0.01). C‐reactive protein level (g/L) (OR = 16.7, 95% CI 2.9–18.9) and neutrophil count (10^9^/L) (OR = 10.1, 95% CI 1.88–21.1) were also confirmed to be predictors (*p* < 0.001). Levels of haemoglobin (g/L) (OR = 16.8, 95% CI 2.4–19.1) and D‐dimer (mg/L) (OR = 5.2, 95% CI 1.2–23.1) were too different to be predictors (*p* < 0.01).

### 3.3 Construction of risk score and nomogram score calculator card

**FIGURE 2:**
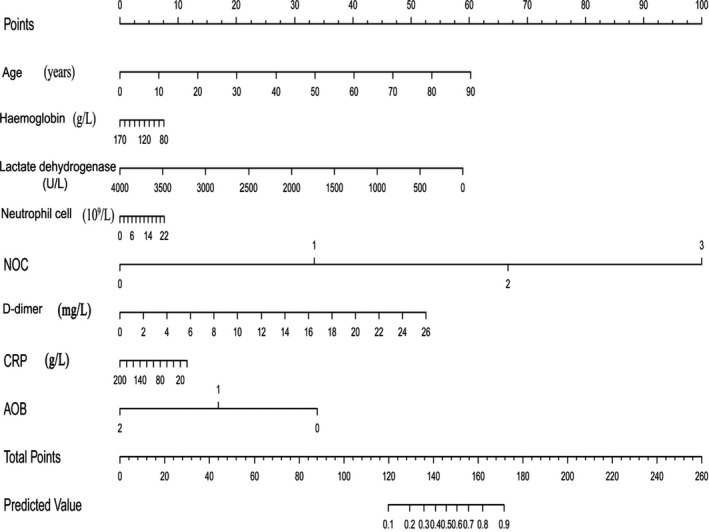
Nomogram risk score plot of predictors of severe coronavirus disease. NOC: number of commodities, AOB: abnormality of biolung

The COVID risk score was constructed based on the coefficients from the logistic model. We used the following formula for the logistic model to calculate the probability and 95% CIs: log probability = 9.571 – [3.284 × C‐reactive protein (g/L)] – [2.992 × neutrophil count (×10^9^/L)] – [4.04 × comorbidities] – [4.102 × bilateral chest CT abnormality] – [1.918 × age] – [0.946 × lactate hydrogenase (U/L)] – [0.391 × D‐dimer (mg/L)] – [0.584 × haemoglobin (g/L)]. A nomogram score calculator card based on eight predictors was developed to allow clinicians to automatically calculate a risk score which can be used to determine the likelihood (with 95% CIs) that a hospitalized patient with COVID‐19 will develop severe illness (Figure 2).

### 3.4 Assessment results of derivation and validation of prediction model for severe COVID‐19 cases

**FIGURE 3:**
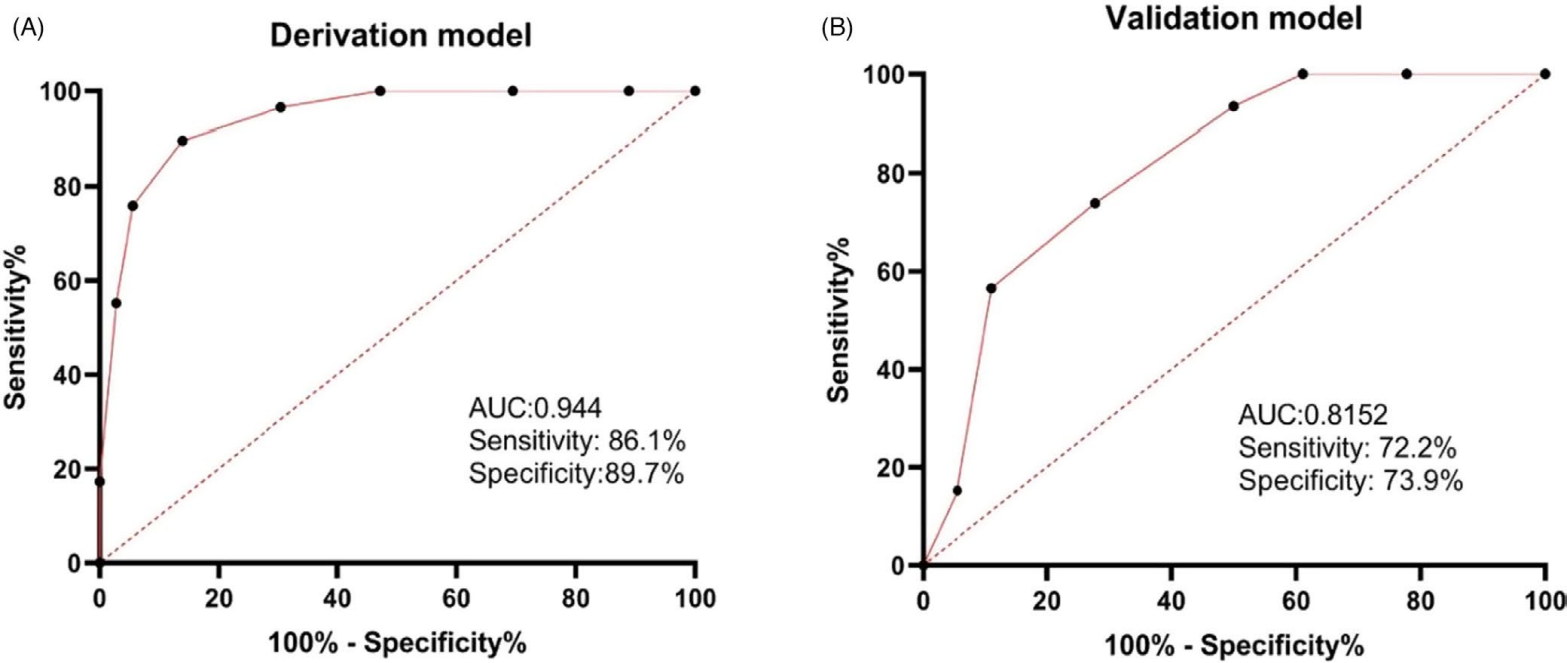
Receiver‐operating characteristic curves based on eight predictors for the predictive model: derivation cohort (3A) and validation cohort (3B). AUC, area under the receiving operating characteristic curve

**FIGURE 4:**
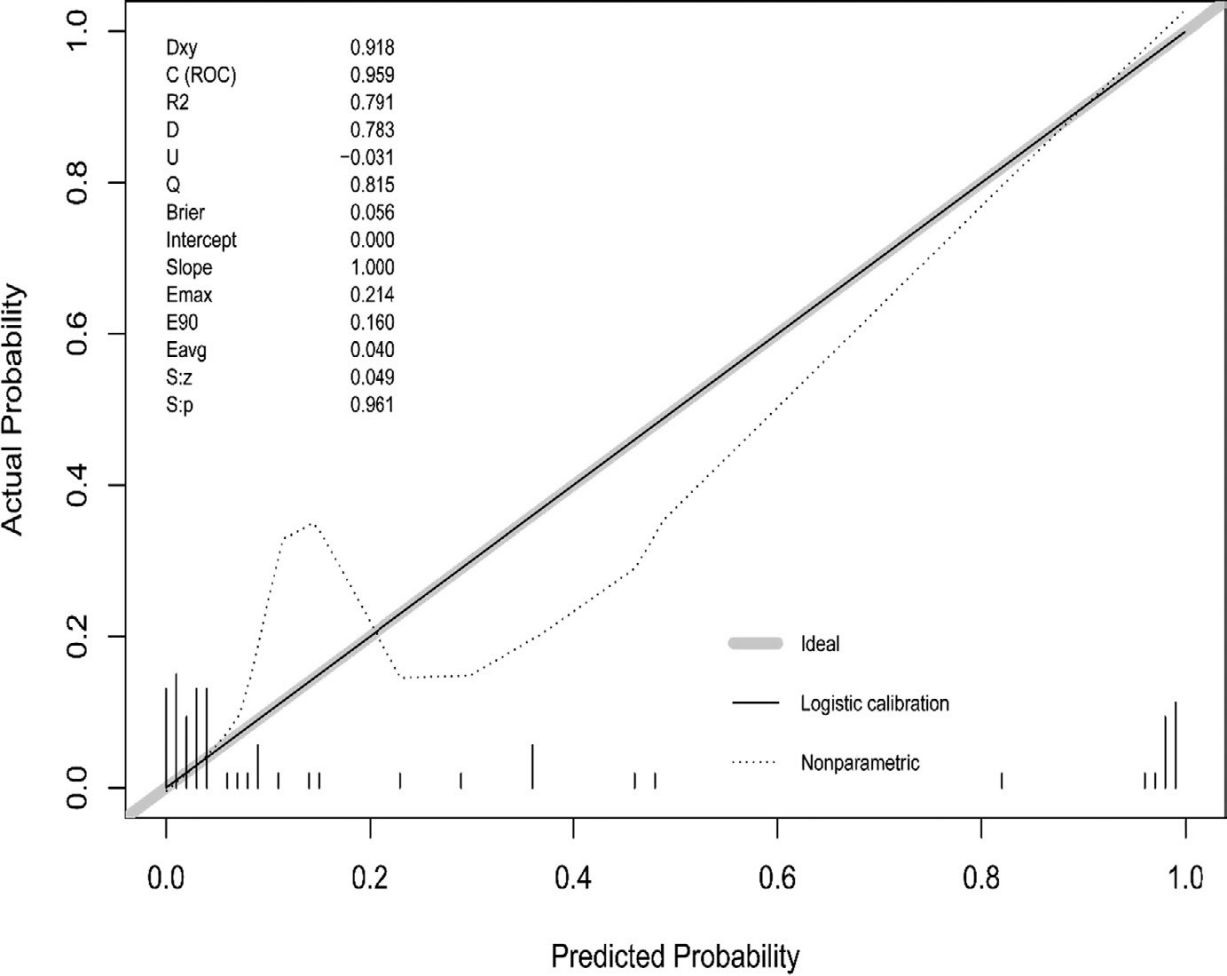
Calibration curve plot of the model in the validation cohort

Taking the predicted probability calculated by the model as the independent variable and the hospitalization outcome as the classification outcome variable, the performance of the prediction model was assessed using the AUC (derivation cohort: 0.944, 95% CI 0.821–0.99; *p* < 0.001 v. validation cohort: 0.8152, 95% CI 0.803–0.97; *p* < 0.001), which indicated that the model had excellent discrimination power (Figure 3A, B). The prediction probability of severe COVID‐19 shown in the calibration curve was in good agreement with the actual probability, and the results suggested that the R‐square on the verification model was 0.791, and the Brier index was 0.056, which indicated that the model had excellent accuracy (Figure 4).

## 4 DISCUSSION

We identified clinical predictors of severe COVID‐19 and developed a prediction model to identify the development of critical illness among hospitalized patients with COVID‐19. The performance of this prediction model was satisfactory, with an accuracy rate of 87.7%. Clinicians can use this model at admission to obtain an early estimate of an individual's risk of developing critical illness. Generally, the eight variables required to calculate the risk probability of developing critical illness are readily available at first hospital admission, and the model‐based score card is easy to use. If the patient's estimated risk for critical illness is low, the clinician may choose to monitor the patient, whereas high‐risk patients would require aggressive treatment, ICU admission or management using ventilators. Previous studies have found several variables to be predictors of severe COVID‐19‐related illness, including older age, higher numbers of comorbidities and imaging abnormalities and higher lactate dehydrogenase levels, all of which were associated with a higher risk of developing acute respiratory distress syndrome in patients with COVID‐19.^9^, ^10^ In the current study, however, we found that lower haemoglobin levels and higher D‐dimer and C‐reactive protein levels were associated with a higher risk of severe COVID‐19. Additionally, lower T‐lymphocyte count and increased IL‐6 level were associated with a higher risk of severe COVID‐19 in the derivation cohort. However, owing to the absence of these two laboratory variables in the validation model, the relevant tests could not be conducted in the study hospitals; therefore, we did not include these two variables in the validation model. To the best of our knowledge, this is the first study to report the combination of these five laboratory variables to predict the severity of COVID‐19. The findings of these laboratory predictors suggest that patients with severe cases of COVID‐19 have a high inflammatory response and low levels of cellular immune dysfunction and coagulopathy. This indicates that these laboratory predictors can be used to diagnose severe cases of COVID‐19 early, so that clinicians can control the cytokine storms and immune cell exhaust of severe cases during treatment.^11^, ^12^

The limitation of this study is its small sample size. The data used in developing the predictive model were entirely from central China, which could potentially limit the global generalizability of the results. Additional validation studies of COVID‐19 predictors should be conducted in regions outside central China.^8^ Moreover, the derivation and validation cohorts were not grouped randomly due to some laboratory parameters could not be conducted in four municipal hospitals.

This study identified the predictors of severe COVID‐19. We also developed a risk score card to estimate, at admission, the risk of developing critical illness among patients with COVID‐19; this was based on eight variables that are commonly measured upon hospital admission. Estimating the risk of critical illness could help in the early identification of patients who are likely to develop severe illness, ensuring appropriate treatment delivery and optimal use of medical resources. In the future, we intend to explore the possibility of developing an online calculator for the rapid and convenient assessment of the risk of developing severe COVID‐19 illness in patients on admission.

## CONFLICT OF INTEREST

The authors declare that there is no conflict of interest regarding the publication of this article.

## AUTHOR CONTRIBUTIONS

YY and QZ designed the study, analysed the data and prepared the article. JZ, JL, KG, YW and JC contributed to the collection and interpretation of the laboratory and clinical data. NJ and WY, BW, BM and QZ were involved in the project management and organizational work. BM and JZ collected data. YY reviewed the article. All authors have read and approved the final version of the article.

## CODE AVAILABILITY

Not applicable.

## CONSENT TO PARTICIPATE

The need for written informed consent was waived because de‐identified retrospective data were used.

## Supporting information

Figure S1

Figure S2

## Data Availability

The data sets used during the current study are available from the corresponding author on reasonable request.
