# Supplementary material for: Early predictors of severe COVID‐19 among hospitalized patients
Source: J Clin Lab Anal. 2021 Dec 23;36(2):e24177. doi: 10.1002/jcla.24177 (PMC8841178; doi:10.1002/jcla.24177)
Supplement: Supplementary file 1 — Figure S1 [file JCLA-36-e24177-s001.docx]

# Supplementary file 1

# Early predictors of severe COVID-19 among hospitalised patients

Qiongrui Zhao, Youhua Yuan^2*^, Jiangfeng Zhang^3*^, Jieren Li^3^, Wei Li^4^, Kunshan Guo^5^, Yanchao Wang^6^, Juhua Chen^7^, Wenjuan Yan^2^, Baoya Wang^2^, Nan Jing^2^, Bing Ma^2^, Qi Zhang^2^

*Corresponding author: Youhua Yuan; E-mail: [yyhnice@163.com](mailto:yyhnice@163.com); Department of Clinical Microbiology, Henan Provincial People’s Hospital, People’s Hospital of Zhengzhou University, and People’s Hospital of Henan University, Zhengzhou 450003, Henan, China

**A**


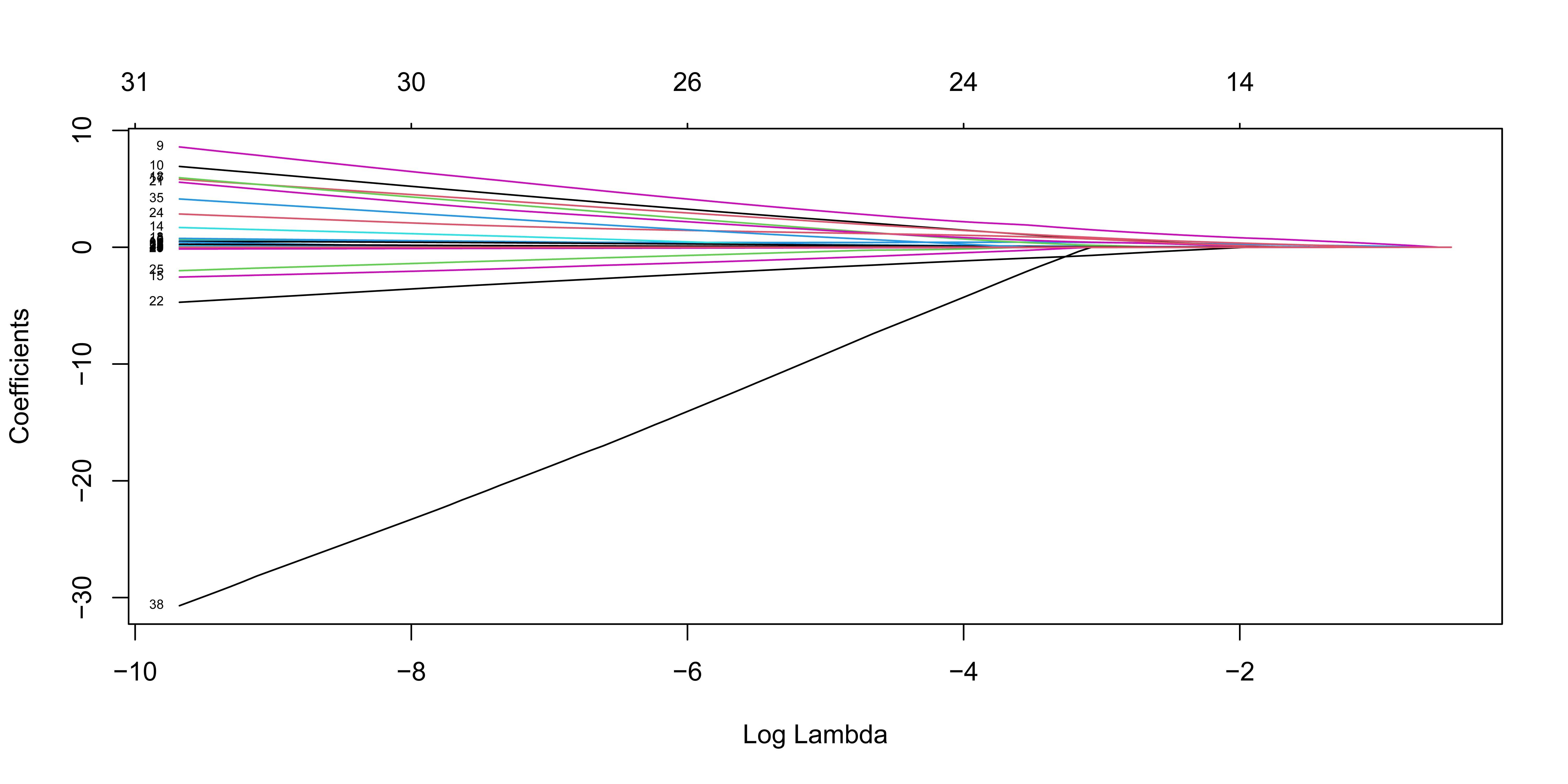


**B**


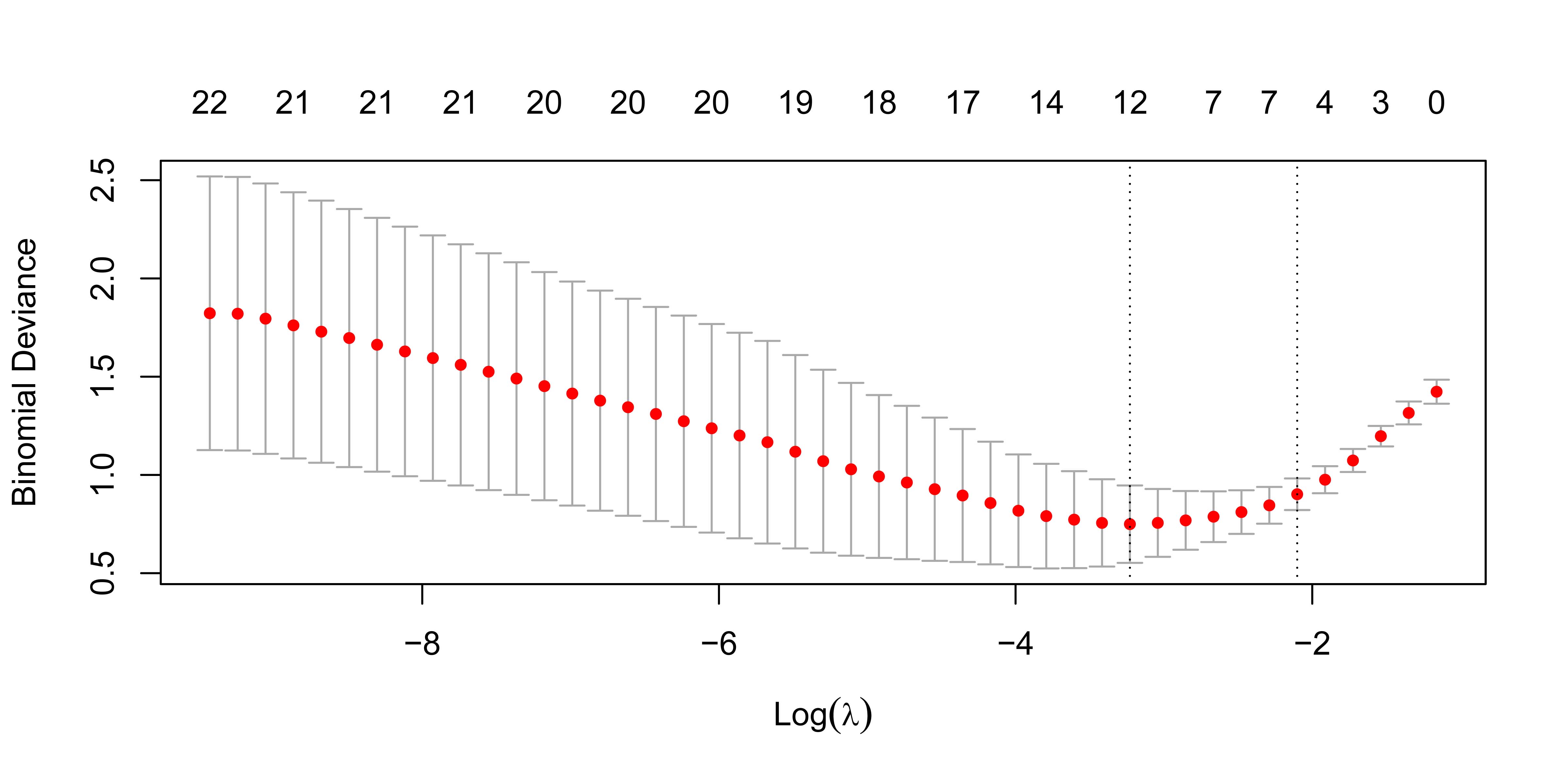


**Fig. S1** Feature selection using the least absolute shrinkage and selection operator (LASSO) binary logistic regression model. (A) LASSO coefficient profiles of the 50 baseline features. (B) Tuning parameter (λ) selection in the LASSO model used 10-fold cross-validation via minimum criteria
