# Supplementary material for: Early predictors of severe COVID‐19 among hospitalized patients
Source: J Clin Lab Anal. 2021 Dec 23;36(2):e24177. doi: 10.1002/jcla.24177 (PMC8841178; doi:10.1002/jcla.24177)
Supplement: Supplementary file 2 — Figure S2 [file JCLA-36-e24177-s002.docx]

# Supplementary file 2

# Early predictors of severe COVID-19 among hospitalised patients

Qiongrui Zhao, Youhua Yuan^2*^, Jiangfeng Zhang^3*^, Jieren Li^3^, Wei Li^4^, Kunshan Guo^5^, Yanchao Wang^6^, Juhua Chen^7^, Wenjuan Yan^2^, Baoya Wang^2^, Nan Jing^2^, Bing Ma^2^, Qi Zhang^2^

*Corresponding author: Youhua Yuan; E-mail: [yyhnice@163.com](mailto:yyhnice@163.com); Department of Clinical Microbiology, Henan Provincial People’s Hospital, People’s Hospital of Zhengzhou University, and People’s Hospital of Henan University, Zhengzhou 450003, Henan, China


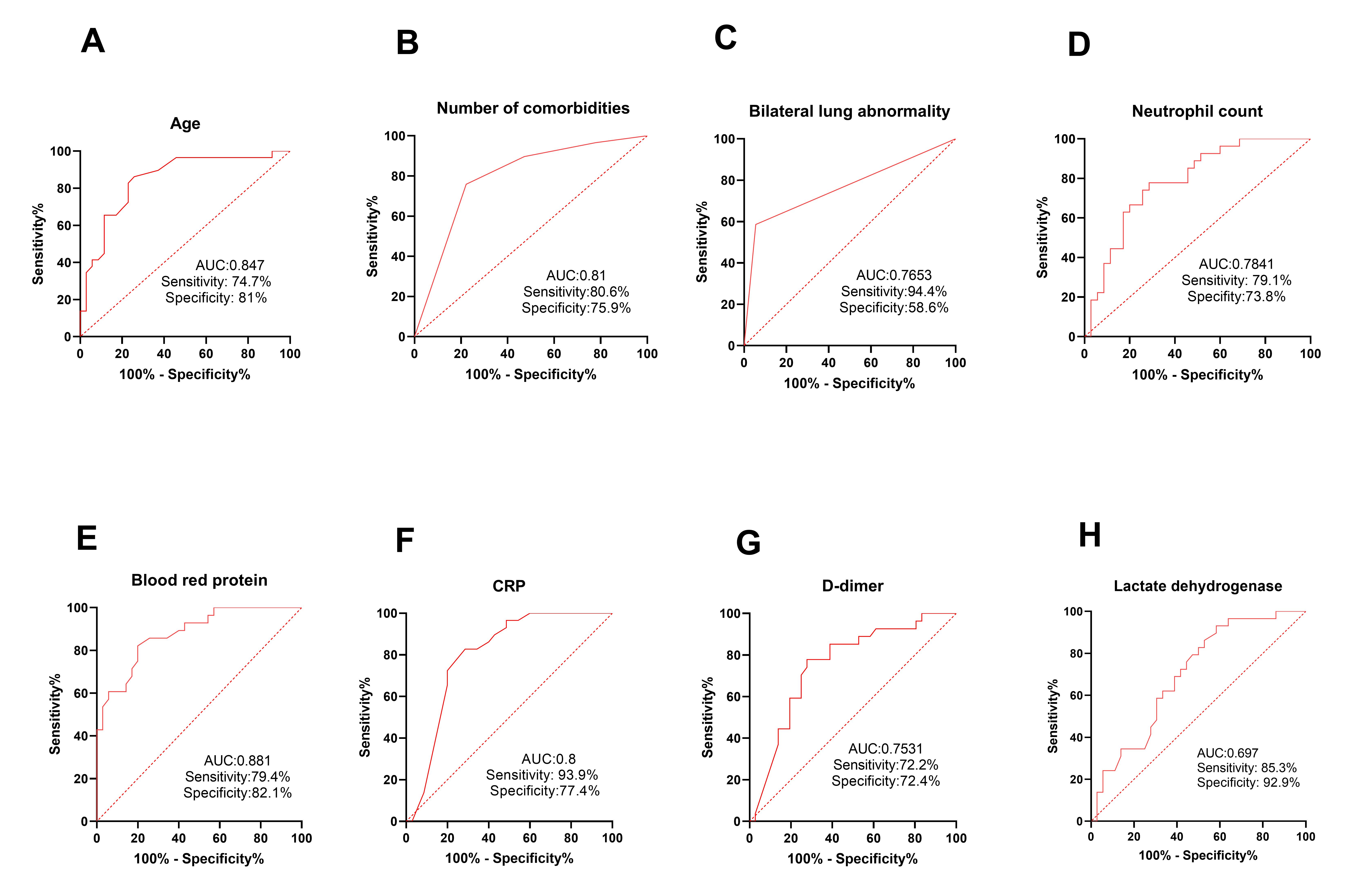


**Fig. S2** Receiver operating characteristic curves of eight predictors in the derived cohort
